# New Insights on Tools for Detecting β-Tubulin Polymorphisms in *Trichuris trichiura* Using rhAmp^TM^ SNP Genotyping

**DOI:** 10.3390/ani14111545

**Published:** 2024-05-23

**Authors:** Julia Rivero, Cristina Cutillas, Rocío Callejón

**Affiliations:** Department of Microbiology and Parasitology, Faculty of Pharmacy, University of Seville, 41012 Seville, Spain; jrfernandez@us.es (J.R.); cutillas@us.es (C.C.)

**Keywords:** anthelmintic resistance, *Trichuris trichiura*, β-tubulin gene, rhAmp^TM^ SNP genotyping, phylogeny

## Abstract

**Simple Summary:**

Soil-transmitted worm infections, usually treated with benzimidazoles, can develop resistance due to genetic variations in a specific gene called β-tubulin isotype 1. This study aimed to create a new, quick, and accurate way to identify these genetic variations. We designed a test to spot changes in certain parts of the β-tubulin gene of *Trichuris trichiura*, the worm causing the infection. By using two different primers, we could distinguish between different genetic types at specific positions in the gene. We tested our method on samples from captive primates and found it to be reliable. Additionally, we explored whether the β-tubulin gene could be useful as a marker in genetic studies. Our tests worked well when we used them on samples from the field. However, we did not find any of the expected genetic variations in the worms or eggs we examined. Instead, all the samples showed the same genetic type. Despite this, our analysis of the β-tubulin gene confirmed the close relationship between *T. trichiura* and a related *Trichuris suis* species, which suggests that this gene could be valuable for understanding their evolutionary history.

**Abstract:**

Soil-transmitted helminth (STH) infections, commonly treated with benzimidazoles, are linked to resistance through single nucleotide polymorphisms (SNPs) at position 167, 198, or 200 in the β-tubulin isotype 1 gene. The aim of this study was to establish a novel genotyping assay characterized by its rapidity and specificity. This assay was designed to detect the presence of SNPs within the partial β-tubulin gene of *Trichuris trichiura*. This was achieved through the biallelic discrimination at codons 167, 198, and 200 by employing the competitive binding of two allele-specific forward primers. The specificity and reliability of this assay were subsequently confirmed using *Trichuris* samples isolated from captive primates. Furthermore, a molecular study was conducted to substantiate the utility of the β-tubulin gene as a molecular marker. The assays showed high sensitivity and specificity when applied to field samples. Nevertheless, none of the SNPs within the β-tubulin gene were detected in any of the adult worms or eggs from the analyzed populations. All specimens consistently displayed an SS genotype. The examination of the β-tubulin gene further validated the established close relationships between the *T. trichiura* clade and *Trichuris suis* clade. This reaffirms its utility as a marker for phylogenetic analysis.

## 1. Introduction

Worldwide, soil-transmitted helminth (STH) infections are among the most common infections that can cause serious harm to human health. It is estimated 1.5 billion people, accounting 24% of the global population, are infected with these parasites, with a higher prevalence observed among preschool and school-age children. The main STH species are *Ascaris lumbricoides* (the roundworm), *Trichuris trichiura* (the whipworm), and *Necator americanus* and *Ancylostoma duodenale* (hookworms). These infections are transmitted through eggs found in human feces, which contaminate soil in regions with inadequate sanitation. This mainly affects impoverished and marginalized communities in tropical and subtropical areas, where the access to clean water, sanitation, and hygiene is limited [1].

The World Health Organization (WHO) has developed a strategy to control the infection of STHs. This strategy aims to regulate morbidity through the periodic treatment, known as preventive chemotherapy, of individuals at risk residing in endemic areas. The objective is to reduce and sustain low infection intensity and protect against morbidity by implementing large-scale mass drug administration programs. WHO recommends treatment with benzimidazoles (BZs) such as albendazoles and mebendazoles due to their effectiveness, affordability, and ease of administration by non-medical personnel [1]. BZs are known to exert their action by blocking the microtubule functions of parasites. This is achieved through the inhibition of β-tubulin polymerization in microtubules, leading to the subsequent inhibition of glucose uptake and transport. As a result, the parasites experience a deficiency of glycogen [2]. However, several studies suggest that the therapeutic efficacy of BZ against trichuriasis is progressively diminishing over time. This decline is believed to be partially attributed to the emergence of anthelminthic drug resistance (AR) [3,4,5], which develops because of prolonged and extensive reliance on BZ anthelmintics over an extended period of time [6]. Furthermore, the drugs are administered in single doses, and while this approach is operationally practical, it does not achieve 100% efficacy [4,7,8,9]. Therefore, this practice of administering suboptimal doses extensively over a prolonged period may contribute to the selection and development of AR. Additionally, periodic treatment has the potential to select for subpopulations of parasites that are resistant to the drugs [10,11,12]. Moreover, there are only a limited number of anthelmintic drugs that have been approved for the treatment of STH infections in humans [13,14].

Single nucleotide polymorphisms (SNPs) have been extensively employed for gene identification. Achieving allelic discrimination for a single SNP with a high degree of reliability and flexibility is of utmost importance for the precise detection of advantageous genes associated with specific SNP sites [15]. BZ resistance in *T. trichiura* is attributed to SNPs in the β-tubulin isotype 1 gene, specifically at codon 167, codon 200 (TTC > TAC), or codon 198 (GAG > GCG) [16,17,18,19,20]. In addition, the frequency of SNPs at codon 200 and 198 was found to increase following treatment, and it was significantly higher in individuals who exhibited a poor response to BZ compared to those who responded well [19]. To maintain the advantages of mass drug administration programs, it is crucial to have tools that can facilitate the large-scale detection of BZ resistance in human STHs. The current challenge of limited detection of phenotypic resistance may be attributed to multiple factors, including the absence of reliable and sensitive methods to monitor resistance genotypes before and after BZ treatment [21], a low frequency of resistance alleles, and the probability that BZ resistance is recessive, as in veterinary parasites [11].

To date, various platforms have been developed for genotyping individual SNPs. These include Kompetitive Allele Specific PCR (KASP) [22,23], RNase H2 enzyme-based amplification (rhAmp) [24], TaqMan [25], and semi-thermal asymmetric reverse PCR (STARP) [26]. Likewise, PCR-based methods, such as real-time PCR (RT-PCR), pyrosequencing, and genotyping assays using the SmartAmp2 method, have been developed for the detection of putative BZ resistance SNPs in human STH [17,18,27,28].

The main objectives of this study were (i) to develop a new genotyping assay, rhAmp^TM^ SNP genotyping, for the screening of β-tubulin SNPs in *T. trichiura*; (ii) to assess the presence of BZ resistance-associated SNPs at positions 167, 198, and 200 within the β-tubulin gene in various populations of *T. trichiura* obtained from non-human primates (These SNPs are likely associated with BZ resistance in *Trichuris* spp.); and (iii) to conduct a molecular investigation aiming to validate the β-tubulin gene as a molecular marker applicable across different *Trichuris* spp. This validation was carried out to infer phylogenetic relationships between different clades of *Trichuris* spp. and detect the emergence of AR, thus gaining insights into its distribution among distinct clades.

## 2. Materials and Methods

### 2.1. Ethics Statement

This study did not require the approval of an ethics committee. Whipworms and eggs were isolated from stool and cecum samples from various vertebrate animal hosts. These animals were housed in zoological gardens and slaughterhouses in Spain and maintained with good animal practices.

### 2.2. Collection Samples

In this study, we have formulated two distinct sections. The first section is based on genotyping assays, for which we utilized *T. trichiura* samples, both eggs and adults, which were collected from different primate hosts. The primate host species analyzed were the Barbary macaque (*Macaca sylvanus*) and patas monkey (*Erythrocebus patas*), from Zoo Castellar (Cádiz, Spain), the vervet monkey (*Chlorocebus aethiops*) from Selwo Aventura (Málaga, Spain), and the Guinea baboon (*Papio papio*) from Parque de la Naturaleza de Cabárceno (Cantabria, Spain) (Table 1). The second section is centered on a phylogenetic analysis, also utilizing the previously mentioned samples, in addition to adult samples from suids (*Sus scrofa domestica*) from slaughterhouses in Seville and Huelva (Spain) and porcupine (*Hystrix cristata*) from Bioparc Fuengirola in Malaga, Spain (Table 1).

Specimens were isolated from stool samples after treatment or collected from the cecum post-mortem and, consequently, washed in saline solution (0.9% *w*/*v*), and separately frozen at −20 °C until further analysis.

Sheather’s sugar solution was used for egg concentration [29], and then, they were embryonated at 32 °C for 3 to 4 weeks with potassium dichromate solution (0.2% *w*/*v*) to provide moisture to the medium and to prevent the growth of fungi and bacteria [30].

### 2.3. Molecular Analysis

#### 2.3.1. DNA Extraction

Whipworm identification and morpho-biometric analysis were performed in previous studies [31,32]. According to the manufacturer’s protocol, total genomic DNA from samples (adult worms and batches of eggs) were extracted using DNeasy Blood and Tissue Kit (Qiagen, Hilden, Germany). To assess the quality of the extractions, 0.8% agarose gel electrophoresis infused with SYBR^TM^ Safe DNA gel stained with 2% *w*/*v* Tris-Borate-EDTA (TBE) was used.

#### 2.3.2. Genotyping by rhAmp SNP Assays

In this study, to carry out the genotyping analysis, we used a dual enzyme chemistry technology called rhAmp^TM^ SNP genotyping [33]. This technology is based on the RNase H2-dependent polymerase chain reaction (rhPCR) and universal reporters [24]. Three SNPs from β-tubulin partial gene (codon 167, 198, and 200) were selected for the rhAmp assays. The selected SNPs with corresponding flanking sequences were submitted to the rhAmp^TM^ Genotyping Design Tool at IDT (Integrate DNATechnologies, IDT; https://eu.idtdna.com/site/order/designtool/index/GENOTYPING_PREDESIGN (accessed on 12 February 2024)) and based on the strength of the thermodynamics, the highest ranked assays were retained. For primer design, flanking sequences shorter than 50 bp (base pairs) were extended up to 50–60 bp based on the *T. trichiura* reference β-tubulin sequence at the probe target sites to meet the technical requirements (Table 2). For each assay, rhAmp used two allele-specific primers and a locus-specific primer. For each SNP, synthetic gBlocks^TM^ Gene fragments were used as known genotype controls during assays, where one represented the wild type (WT) and the other, the mutant allele (MA), and furthermore, both were mixed in an equal molar ratio to represent the heterozygous genotype (Table 3). 

SNP genotyping assays were performed using 0.25 μL of rhAmp SNP Assays (20X), 2.65 μL of combined rhAmp Genotyping Master Mix (2X) and rhAmp Reporter Mix (40X), 0.10 μL of nuclease-free water and 2 μL of sample DNA, and 2 μL of control template (gBlocks fragments controls) or 2 μL of nuclease-free water (for no-template control reactions). Reactions were run on the CFX Connect Real-Time PCR Detection System (Bio-Rad Laboratories, Hercules, CA, USA), and analyses were carried out using CFX Maestro Software version 2.3 (Bio-Rad, Hercules, CA, USA). The thermal conditions were 95 °C for 10 min, followed by 40 cycles at 95 °C for 10 s, 60 °C for 30 s, and 68 °C for 20 s per the published protocol (www.idtdna.com/rhAmp-SNP-protocol (accessed on 29 February 2024).

The bi-allelic specificity of the rhAmp assays was provided by two probes, one labelled with Amidite-fluorescein (FAM^TM^) dye and the other with Hexachloro-fluorescein (HEX^TM^) dye (Table 4). These different dye reporters were independently detected with excitation sources and emission filters at the respective wavelengths. A total of 91 *Trichuris* genomic DNA samples were quantified. Hence, each specimen was called resistant (RR), susceptible (SS), and heterozygous (RS) in relation to the melting temperature obtained.

#### 2.3.3. PCR and Sequencing

In the samples analyzed in the present study, the partial molecular marker gene β-tubulin was amplified by a polymerase chain reaction (PCR) using a thermal cycler (Eppendorf AG and sequenced. (Hamburg, Germany). The primers and PCR conditions were previously described by Hansen et al. [34]. Amplification reactions consisted of 5 μL (10 μM) of each primer, 5 μL of template DNA (50 ng/μL,) 25 μL of GoTaq G2 Green Master Mix, and nuclease-free water up to 50 μL. A negative (no-template DNA) control sample and a positive DNA control sample were included in each PCR reaction. PCR products were visualized on agarose gels (0.8%). Subsequently, bands were eluted and purified using the Wizard SV Gel and PCR Clean-Up System Kit (Promega, WI, USA). Once purified, PCR products were concentrated and sequenced in both directions by Stab Vida (Lisbon, Portugal).

#### 2.3.4. Phylogenetic Studies

Accession numbers obtained in this study are available in the GenBank database (Table 1). To analyze the relationships among the different *Trichuris* species, additional sequences from GenBank database were included in the alignments (Supplementary Table S1).

The aligned nucleotide dataset was obtained by the MUSCLE alignment method in MEGA (Molecular Evolutionary Genetics Analysis) version 11 [35]. Moreover, the number of nucleotide differences per sequence was calculated to evaluate the identity among *Trichuris* β-tubulin partial sequences by Compute Pairwise Distances based on the number of differences method of MEGA11 [35].

All phylogenetic trees were inferred by two different methods, Maximum Likelihood (ML) and Bayesian Inferences (BIs). To generate the ML tree, PhyML 3.0 package [36] was used, and for the BI tree, MrBayes v3.2.6 [37] was used. To resolve the best-fit substitution model for the nucleotide dataset jModelTest [38] was employed, and the models of evolution were determined in agreement with Akaike Information Criterion [36,39]. Bootstrapping (heuristic option) of more than 1000 replications was used to examine the topology support to assess the relative reliability of the clades, and the Bayesian Posterior Probabilities (BPPs) comprised the percentage converted. Standard deviation of split frequencies was used to determine if the number of generations completed was adequate. Each dataset was run for 10 million generations, and the chain was sampled every 500 generations. In addition, trees from the first million generations were discarded based on an assessment of convergence. Empirically, during the burn, the examination of the log-likelihood values of the chain was carried out.

## 3. Results

### 3.1. SNP Genotyping Assays

rhAmp^TM^ SNP Genotyping were completely optimized at codon 167, 198, and 200 of the β-tubulin gene in *T. trichiura* and were able to detect the presence or absence of the WT and MA genotypes. 

Before real-time analysis, for each SNP analyzed, standard peaks were created for RR (homozygote-resistant), SS (homozygote-susceptible), and RS (heterozygote) using WT, MA, and heterozygote DNA samples obtained commercially from IDT. 

**Figure 1:**
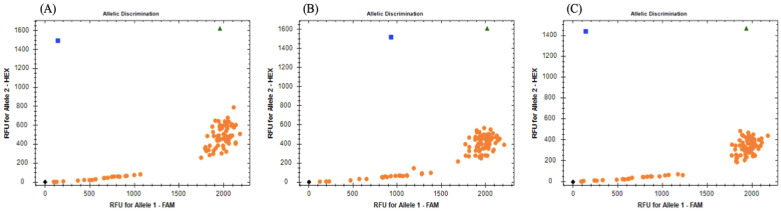
Allelic discrimination plots obtained for rhAmp^TM^ SNP genotyping assays targeting the three SNPs: (**A**) codon 167; (**B**) codon 198; (**C**) codon 200. Homozygous genotypes are represented by orange dots and blue squares, where the orange dots correspond to an SS and the blue squares to an RR, the green triangles represent heterozygous genotypes, and the black rhombus on the bottom left of the plot are no-template controls.

For each point mutation site, the melting peaks obtained from RT-PCR were analyzed and determined as RR, SS, and RS as reported by the specific Tm. Ninety-one Trichuris samples were collected from non-human primates, and genotyping assays by RT-PCR revealed the different dots, homozygote and heterozygote, obtained in the present work (Figure 1). Positive (WT, MA, and heterozygote) and negative controls were always included, and no amplification in negative controls was observed. 

Out of the three analyzed SNPs in the partial β-tubulin gene, all specimens exhibited SS (100%). However, for codon 168, low signal intensity was observed in all twenty-one egg batch samples for both fluorophores. For codon 198, only four egg batch samples showed low signal intensity, and for codon 200, seven egg batch samples displayed low signal intensity. To ensure the accuracy of the results, all experiments were repeated twice. In concordance with these results, the resistance allele frequency (RAF) was 0.0 for each SNP studied.

### 3.2. Molecular Analysis

**Figure 2:**
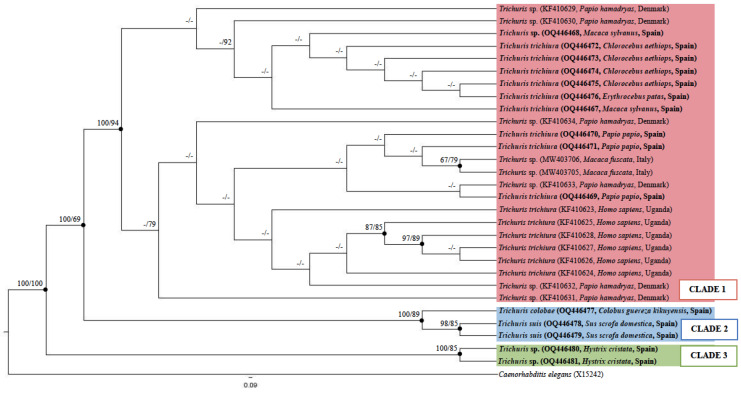
Phylogenetic tree inferred based on the molecular marker β-tubulin partial gene of *Trichuris* species using ML method. Bayesian Posterior Probabilities (BPPs) are listed first, followed by ML bootstrap values (BVs) of clades, for clade frequencies exceeding 60%.

The phylogenetic tree inferred by ML and BI methods revealed three main clades (Figure 2). Clade 1 consisted of sequences from both, *Trichuris* sp. and *T. trichiura*, isolated from humans and non-human primates (100 BPP and 94% ML BV). In the BI analysis, the sequences were in polytomy, but the support for different subclades was not strong based on ML methods. Clade 2 consisted of sequences from *Trichuris colobae* and *Trichuris suis* (100 BPP and 89% ML BV), which were grouped into two distinct and well-supported subclades. Further, clade 3 consisted of sequences from *Trichuris* sp. isolated from *H. cristata* that were strongly supported (100 BPP and 85% ML BV) (Figure 2). 

The results obtained were consistent with the identity analysis, where sequences clustered within the same clade displayed an inter-populational similarity percentage of over 98.7% for clade 1, ranging from 98 to 100% for clade 2, and 100% for clade 3 (Supplementary Table S2a,b). The similarity percentage between clade 1 and clade 2 ranged from 91.3 to 92.3%, between clade 1 and clade 3, 81.8–82.9%, and between clade 2 and clade 3, 82.9–83.1% (Supplementary Table S2a,b). 

Phylogenetic analysis revealed a sister relationship between clade 1, which included *T. trichiura* and *Trichuris* sp. isolated from humans and non-human primates, and clade 2, which comprised *T. suis* and *T. colobae* (100 BPP and 69% ML BV). Likewise, both clades remained separate from *Trichuris* sp. from porcupine (Figure 2). 

## 4. Discussion

STHs have been widely treated with mass drug administration, a highly effective approach in reducing helminth-related morbidity by limiting transmission within endemic communities. However, while this strategy brings numerous advantages to the population, it may also lead to unintended consequences, such as the gradual reduction in treatment effectiveness [40,41]. Consequently, with the expansion of drug donations, the emergence of AR becomes increasingly probable. This decline in treatment efficacy has been particularly concerning in veterinary parasites, where certain nematode species have demonstrated high levels of resistance to drugs, including BZ [11,12,42,43,44]. Moreover, in these helminths, SNPs in the β-tubulin isotype 1 gene have been associated with resistance to BZ [10,11,12].

Several tests have been proposed to identify mutations related to BZ resistance in helminths [19,45,46]. The SmartAmp2 constituted an alternative approach utilized. This method streamlines the process by implementing a single-step protocol, enabling enhanced expediency, showcasing distinct advantages over conventional PCR. Additionally, it has demonstrated efficacy in the investigation of diverse STHs identified in fecal samples. Nevertheless, utilizing this technique, the optimization of all necessary SNP detections proved unattainable, as the identification of codon 167 in *T. trichiura* was unsuccessful [28]. Diarra et al. [19] conducted an evaluation of the SNPs in codon 200 in A. lumbricoides using pyrosequencing, which necessitates specialized equipment. Another method employed was the RFLP-PCR, which is considered simpler and more sensitive but has certain limitations, such as cases where the DNA sequences are not recognized by commercially available restriction enzymes or possess multiple recognition sites for a single enzyme [45]. In contrast, Furtado et al. [46] employed ARMS-PCR, a technique that solely requires a conventional thermocycler, offering a straightforward and immediate result. Broccanello et al. [47] conducted a comparison of the accuracy, sensitivity, and costs of TaqMan, KASP, and rhAmp^TM^ SNP Genotyping methods in sugar beet (*Beta vulgaris* L.). The sensitivity test revealed that both TaqMan and rhAmp were able to accurately determine SNP genotypes. In the case of rhAmp^TM^ SNP Genotyping, 24 of the 33 SNPs exhibited 100% concordance with other two technologies. The genotype concordance with both technologies for the remaining nine targets exceeded 99%.

This study represents the initial endeavor to establish rhAmp assays for genotyping SNPs in the β-tubulin partial gene of *Trichuris* spp. samples. The primary objective was to develop a rapid and highly sensitive method capable of discriminating between different alleles in *Trichuris* samples, while also determining the genotyping frequencies. The developed rhAmp methods proved effective in accurately identifying the three different genotypes. Sequencing of the PCR products further confirmed the high conservation of the region flanked by the primers (PCR) and probes (rhAmp) which also contained the mutation. 

None of the analyzed SNPs were found in our examined samples. Hence, the prevalence observed in our study was found to be lower compared to previously reported prevalence rates for other parasites. For instance, the prevalence of A. lumbricoides was reported to be 0.5% [46], while *Haemonchus contortus* showed a much higher prevalence of 74% [48]. Nonetheless, certain studies examining theses SNPs in parasites of veterinary significance, such as *P. equorum* and *Ascaridia galli*, have failed to detect these alterations, even in parasite populations subjected to frequent treatments throughout the year [49,50,51]. However, our findings are consistent with those obtained by Hansen et al. [34], wherein none of the 27 adult worms and 39 egg batched of *Trichuris* from human analyzed, or the 49 adult worms of *Trichuris* samples from baboons, exhibited mutations in any of the analyzed SNPs. This suggests that the identified SNPs may not be the primary mechanism responsible for BZ resistance in these nematode species. 

The methodology proposed here offers a robust tool for screening the emergence of anthelmintic resistance mutations in populations of parasitic nematodes. Furthermore, it represents a rapid, highly sensitive, and specific technique that obviates the need for PCR amplification, a thermocycler, and electrophoresis. 

Additionally, according to previous phylogenetic studies carried out by other authors, which were based on both nuclear and mitochondrial markers and focused on *Trichuris* species, two main clades have been identified. These clades have been previously referred to as the “*T. trichiura* lineage”, which parasitizes humans and non-human primates, and the “*T. suis* lineage”, which infects suids and other primates, such as *Colobus guereza kikuyensis* or *Papio ursinus* [31,32]. Furthermore, various authors have reported the hypothesis that a complex whipworm species may exist in primates, suggesting that different *Trichuris* species infect both primates and humans [32,52,53,54,55,56]. In addition, our results align with those obtained by Rivero et al. [57], where, based on nuclear and mitochondrial markers as well, the *Trichuris* sequences isolated from porcupines were distinct from the rest of the analyzed sequences, remaining isolated from the rest of the sequences in the obtained tree. As a result, the results obtained confirmed the sister relationship between clade 1 and clade 2 as identified in the phylogenetic tree. This finding corroborates the utility of β-tubulin as an additional marker, in addition to those previously described, for the phylogenetic analysis of the *Trichuris* species.

## 5. Conclusions

The current study provides a novel genotyping assay for assessing the prevalence of frequency of AR-associated β-tubulin SNPs at codons 167, 198, and 200 in *T. trichiura*. This investigation has showcased the efficacy of rhAmp methods in accurately discriminating and identifying β-tubulin SNPs within *T. trichiura* adults and eggs. Consequently, rhAmp^TM^ SNP Genotyping can be considered a molecular tool that is both rapid and sensitive for the in vitro assessment of BZ susceptibility in *Trichuris* spp. Moreover, using pooled samples, it can be proposed as a cost-effective method, offering immediate practical advantages in the field. 

Furthermore, this study provides further insights into the phylogeny of β-tubulin in *Trichuris* spp., reaffirming the previously established close relationship between the *T. trichiura* clade and *T. suis* clade. Thus, we affirm its usefulness as a marker for phylogenetic analysis. 

## Figures and Tables

**Table 1:** Sequences of *Trichuris* spp. species obtained in the present study based on β-tubulin partial gene including sample ID, host species, geographical origin, GenBank accession numbers, length, and G + C content.

Species	Sample ID	Adult/Eggs	Host Species/Geographical Origin	Accession Number	Length (bp)	G + C Content (%)
*Trichuris trichiura*	TMSM9	Adult	*Macaca sylvanus*/Spain	OQ446467	445	44.94
*Trichuris* sp.	TMSF10	Adult	*Macaca sylvanus*/Spain	OQ446468	445	44.94
*Trichuris trichiura*	TPPM1	Adult	*Papio papio*/Spain	OQ446469	445	45.39
*Trichuris trichiura*	TPPF1	Adult	*Papio papio*/Spain	OQ446470	445	45.39
*Trichuris trichiura*	TPPF2	Adult	*Papio papio*/Spain	OQ446471	445	45.39
*Trichuris trichiura*	TCAE_1	Eggs	*Chlorocebus aethiops*/Spain	OQ446472	445	44.94
*Trichuris trichiura*	TCAE_2	Eggs	*Chlorocebus aethiops*/Spain	OQ446473	445	44.94
*Trichuris trichiura*	TCAE_3	Eggs	*Chlorocebus aethiops*/Spain	OQ446474	445	44.94
*Trichuris trichiura*	TCAE_4	Eggs	*Chlorocebus aethiops*/Spain	OQ446475	445	44.94
*Trichuris trichiura*	TEPE	Eggs	*Erythrocebus patas*/Spain	OQ446476	445	44.94
*Trichuris colobae*	TCO_901	Adult	*Colobus guereza kikuyensis*/Spain	OQ446477	445	44.94
*Trichuris suis*	TSF1	Adult	*Sus scrofa domestica*/Spain	OQ446478	445	44.72
*Trichuris suis*	TSF2	Adult	*Sus scrofa domestica*/Spain	OQ446479	445	44.72
*Trichuris* sp.	THCF10	Adult	*Hystrix cristata*/Spain	OQ446480	448	47.99
*Trichuris* sp.	THCF15	Adult	*Hystrix cristata*/Spain	OQ446481	448	47.99

**Table 2:** Primers designed for rhAmp SNPs Assays for each codon using rhAmp^TM^ Genotyping Design Tool at IDT.

Codon	*T. trichiura* rhAmp SNP Assays		
	Primer Type	Primer	Sequence
**Codon 167**	Allele-Specific	Primer 1	CCTGACCGAATTATGACAACTTT
	Allele-Specific	Primer 2	CCTGACCGAATTATGACAACTTA
	Locus-Specific	Primer	GCCCCAACGTGAACAGTATCAAA
**Codon 198**	Allele-Specific	Primer 1	GCTTCATTATCTATGCAGAATGTTT
	Allele-Specific	Primer 2	GCTTCATTATCTATGCAGAATGTTG
	Locus-Specific	Primer	GCATGCAACTCTGTCAGTCCA
**Codon 200**	Allele-Specific	Primer 1	AGCGCTTCATTATCTATGCAGAA
	Allele-Specific	Primer 2	AGCGCTTCATTATCTATGCAGAT
	Locus-Specific	Primer	GCATGCAACTCTGTCAGTCCA

**Table 3:** gBlocks^®^ designed for β-tubulin partial gene of *T. trichiura* genotyping corresponding to the three SNPs (codon 167, 198, and 200). The SNPs are indicated in red color and in bold in the sequences.

*T. trichiura* gBlocks^®^ Gene Fragments	Sequence
gBlocks^®^ WT	GAATCGGAAAGCTGCGACTGCCTGCAAGGGTTCCAGTTGACTCATTCCCTCGGCGGCGGAACTGGGAGTGGAATGGGTACGCTTCTGATATCTAAAATTCGGGAAGAGTATCCTGACCGAATTATGACAACTT**T**TAGTGTCGTTCCGTCTCCGAAGGCAAGTTGTTTGATACTGTTCACGTCGTGAACTATCGCCTTTTTAGGTTTCAGATACAGTTGTAGAACCATATAATGCAACTCTGTCAGTCCACCAGTTGGTAGAGAACACGGACG**A**AACA**T**TCTGCATAGATAATGAAGCGCTTTACGATATTTGTTTCCGAACTTTGAAGTTAACAACACCAACTTACGGAGACTTAAATCATTTGGTTTCGGCAACCATGTCTGGAGTAACGACATGCCTACGCTTTCCTGGTCAGTTGAATGCTGATTTGCGGAAGCTGGCAGTC
gBlocks^®^ 167	GAATCGGAAAGCTGCGACTGCCTGCAAGGGTTCCAGTTGACTCATTCCCTCGGCGGCGGAACTGGGAGTGGAATGGGTACGCTTCTGATATCTAAAATTCGGGAAGAGTATCCTGACCGAATTATGACAACTT**A**TAGTGTCGTTCCGTCTCCGAAGGCAAGTTGTTTGATACTGTTCACGTCGTGAACTATCGCCTTTTTAGGTTTCAGATACAGTTGTAGAACCATATAATGCAACTCTGTCAGTCCACCAGTTGGTAGAGAACACGGACG**A**AACA**T**TCTGCATAGATAATGAAGCGCTTTACGATATTTGTTTCCGAACTTTGAAGTTAACAACACCAACTTACGGAGACTTAAATCATTTGGTTTCGGCAACCATGTCTGGAGTAACGACATGCCTACGCTTTCCTGGTCAGTTGAATGCTGATTTGCGGAAGCTGGCAGTC
gBlocks^®^ 198	GAATCGGAAAGCTGCGACTGCCTGCAAGGGTTCCAGTTGACTCATTCCCTCGGCGGCGGAACTGGGAGTGGAATGGGTACGCTTCTGATATCTAAAATTCGGGAAGAGTATCCTGACCGAATTATGACAACTT**T**TAGTGTCGTTCCGTCTCCGAAGGCAAGTTGTTTGATACTGTTCACGTCGTGAACTATCGCCTTTTTAGGTTTCAGATACAGTTGTAGAACCATATAATGCAACTCTGTCAGTCCACCAGTTGGTAGAGAACACGGACG**C**AACA**T**TCTGCATAGATAATGAAGCGCTTTACGATATTTGTTTCCGAACTTTGAAGTTAACAACACCAACTTACGGAGACTTAAATCATTTGGTTTCGGCAACCATGTCTGGAGTAACGACATGCCTACGCTTTCCTGGTCAGTTGAATGCTGATTTGCGGAAGCTGGCAGTC
gBlocks^®^ 200	GAATCGGAAAGCTGCGACTGCCTGCAAGGGTTCCAGTTGACTCATTCCCTCGGCGGCGGAACTGGGAGTGGAATGGGTACGCTTCTGATATCTAAAATTCGGGAAGAGTATCCTGACCGAATTATGACAACTT**T**TAGTGTCGTTCCGTCTCCGAAGGCAAGTTGTTTGATACTGTTCACGTCGTGAACTATCGCCTTTTTAGGTTTCAGATACAGTTGTAGAACCATATAATGCAACTCTGTCAGTCCACCAGTTGGTAGAGAACACGGACG**A**AACA**A**TCTGCATAGATAATGAAGCGCTTTACGATATTTGTTTCCGAACTTTGAAGTTAACAACACCAACTTACGGAGACTTAAATCATTTGGTTTCGGCAACCATGTCTGGAGTAACGACATGCCTACGCTTTCCTGGTCAGTTGAATGCTGATTTGCGGAAGCTGGCAGTC

**Table 4:** Different excitation and emission spectra for fluorophores.

Dye	λ Excitation Filter (nm)	λ Emission Filter (nm)
FAM^TM^	450	533
HEX^TM^	483	568

## Data Availability

The data presented in this study are available on request from the corresponding author.

## Supplementary Materials

The following supporting information can be downloaded at: https://www.mdpi.com/article/10.3390/ani14111545/s1, Table S1: Sequences of *Trichuris* spp. and outgroups species obtained from GenBank and used for phylogenetic analysis; Table S2a: Intra-specific and inter-specific similarity observed in β-tubulin partial gene sequences in *Trichuris* species isolated from different hosts. Values are given in percentages (%); Table S2b: Continuation of Table S2a. Intra-specific and inter-specific similarity observed in β-tubulin partial gene sequences in *Trichuris* species isolated from different hosts. Values are given in percentages (%).
