# Supplementary material for: New Insights on Tools for Detecting β-Tubulin Polymorphisms in Trichuris trichiura Using rhAmpTM SNP Genotyping
Source: Animals (Basel). 2024 May 23;14(11):1545. doi: 10.3390/ani14111545 (PMC11171370; doi:10.3390/ani14111545)
Supplement: Supplementary file 1 [file animals-14-01545-s001.zip › Supplementary Table S1.pdf]

Table S1. Sequences of *Trichuris* spp. and outgroups species obtained from GenBank and used for phylogenetic analysis.

Species	Host species/ Geographical origin	Accession number
Trichuris trichiura	*Homo sapiens*/Uganda	KF410623
Trichuris trichiura	*Homo sapiens*/Uganda	KF410624
Trichuris trichiura	*Homo sapiens*/Uganda	KF410625
Trichuris trichiura	*Homo sapiens*/Uganda	KF410626
Trichuris trichiura	*Homo sapiens*/Uganda	KF410627
Trichuris trichiura	*Homo sapiens*/Uganda	KF410628
Trichuris trichiura	*Homo sapiens*/Jamaica	AF118385
Trichuris trichiura	*Homo sapiens*/Jamaica	AF034219
Trichuris muris	*Mus musculus*/Edinburgh	BM174835
Trichuris muris	*Mus musculus*/Edinburgh	BF049947
Trichuris vulpis	*Canis lupus familiaris*/ North Carolina	CB189017
Trichuris vulpis	*Canis lupus familiaris*/ North Carolina	CB099433
Trichuris vulpis	*Canis lupus familiaris*/ North Carolina	CB188039
Trichuris sp.	*Macaca fuscata*/Italy	MW403705
Trichuris sp.	*Macaca fuscata*/Italy	MW403706
Trichuris sp.	*Papio hamadryas*/Denmark	KF410629
Trichuris sp.	*Papio hamadryas*/Denmark	KF410630
Trichuris sp.	*Papio hamadryas*/Denmark	KF410631
Trichuris sp.	*Papio hamadryas*/Denmark	KF410632
Trichuris sp.	*Papio hamadryas*/Denmark	KF410633
Trichuris sp.	*Papio hamadryas*/Denmark	KF410634
**Outgroup**		**Accession number**
Caenorhabditis elegans		X15242
