# Supplementary material for: New Insights on Tools for Detecting β-Tubulin Polymorphisms in Trichuris trichiura Using rhAmpTM SNP Genotyping
Source: Animals (Basel). 2024 May 23;14(11):1545. doi: 10.3390/ani14111545 (PMC11171370; doi:10.3390/ani14111545)
Supplement: Supplementary file 1 [file animals-14-01545-s001.zip › Supplementary Table S2a.pdf]

Table S2a. Intra-specific and inter-specific similarity observed in β-tubulin partial gene sequences in *Trichuris* species isolated from different hosts. Values are given in percentages (%).

	OQ446467 *T. trichiura* from *M. sylvanus* TMSM9	OQ446468 *Trichuris* sp. from *M. sylvanus* TMSF10	OQ446469 *T. trichiura* from *P. papio* TPPM1	OQ446470 *T. trichiura* from *P. papio* TPPF1	OQ446471 *T. trichiura* from *P. papio* TPPF2	OQ446472 *T. trichiura* from *C. aethiops* TCAE_1	OQ446473 *T. trichiura* from *C. aethiops* TCAE_2	OQ446474 *T. trichiura* from *C. aethiops* TCAE_3	OQ446475 *T. trichiura* from *C. aethiops* TCAE_4	OQ446476 *T. trichiura* from *E. patas* TEPE	OQ446477 *T. colobae* TCO_901	OQ446478 *T. suis* TSF1	OQ446479 *T. suis* TSF2	OQ446480 *Trichuris* sp. from *H. cristata* TPEF10
OQ446468 *Trichuris* sp. from *M. sylvanus* TMSF10	100													
OQ446469 *T. trichiura* from *P. papio* TPPM1	99,49	99,49												
OQ446470 *T. trichiura* from *P. papio* TPPF1	99,49	99,49	100											

OQ446471 *T. trichiura* from *P. papio* TPPF2	99,49	99,49	100	100										
OQ446472 *T. trichiura* from *C. aethiops* TCAE_1	100	100	99,49	99,49	99,49									
OQ446473 *T. trichiura* from *C. aethiops* TCAE_2	100	100	99,49	99,49	99,49	100								
OQ446474 *T. trichiura* from *C. aethiops* TCAE_3	100	100	99,49	99,49	99,49	100	100							
OQ446475 *T. trichiura* from *C.*	100	100	99,49	99,49	99,49	100	100	100						

aethiops TCAE_4														
OQ446476 *T. trichiura* from *E. patas* TEPE	100	100	99,49	99,49	99,49	100	100	100	100					
OQ446477 *T. colobae* TCO_901	91,82	91,82	91,82	91,82	91,82	91,82	91,82	91,82	91,82	91,82				
OQ446478 *T. suis* TSF1	92,33	92,33	91,82	91,82	91,82	92,33	92,33	92,33	92,33	92,33	97,95			
OQ446479 *T. suis* TSF2	92,33	92,33	91,82	91,82	91,82	92,33	92,33	92,33	92,33	92,33	97,95	100		
OQ446480 *Trichuris* sp. from *H. cristata* TPEF10	82,61	82,61	82,61	82,61	82,61	82,61	82,61	82,61	82,61	82,61	82,86	83,12	83,12	
OQ446481	82,61	82,61	82,61	82,61	82,61	82,61	82,61	82,61	82,61	82,61	82,86	83,12	83,12	100

Trichuris sp. from *H. cristata* TPEF15														
KF410628 *T. trichiura* from *H. sapiens*	98,72	98,72	99,23	99,23	99,23	98,72	98,72	98,72	98,72	98,72	91,82	91,30	91,30	82,61
KF410627 *T. trichiura* from *H. sapiens*	98,98	98,98	99,49	99,49	99,49	98,98	98,98	98,98	98,98	98,98	91,82	91,30	91,30	82,35
KF410626 *T. trichiura* from *H. sapiens*	98,72	98,72	99,23	99,23	99,23	98,72	98,72	98,72	98,72	98,72	91,56	91,05	91,05	82,10
KF410625 *T. trichiura* from *H. sapiens*	99,23	99,23	99,74	99,74	99,74	99,23	99,23	99,23	99,23	99,23	92,07	91,56	91,56	82,35
KF410624 *T. trichiura*	99,23	99,23	99,74	99,74	99,74	99,23	99,23	99,23	99,23	99,23	91,56	91,56	91,56	82,86

from *H. sapiens*														
KF410623 *T. trichiura* from *H. sapiens*	99,49	99,49	100,00	100,00	100,00	99,49	99,49	99,49	99,49	99,49	91,82	91,82	91,82	82,61
MW403705 *Trichuris* sp. from *M. fuscata*	98,98	98,98	99,49	99,49	99,49	98,98	98,98	98,98	98,98	98,98	91,82	91,82	91,82	82,10
MW403706 *Trichuris* sp. from *M. fuscata*	98,72	98,72	99,23	99,23	99,23	98,72	98,72	98,72	98,72	98,72	91,56	91,56	91,56	81,84
KF410634 *Trichuris* sp. *P. hamadryas*	99,49	99,49	100	100	100	99,49	99,49	99,49	99,49	99,49	91,82	91,82	91,82	82,61
KF410633 *Trichuris* sp. *P.*	99,23	99,23	99,74	99,74	99,74	99,23	99,23	99,23	99,23	99,23	91,56	91,56	91,56	82,86

hamadryas														
KF410632 *Trichuris* sp. *P. hamadryas*	99,49	99,49	100	100	100	99,49	99,49	99,49	99,49	99,49	91,82	91,82	91,82	82,61
KF410631 *Trichuris* sp. *P. hamadryas*	99,74	99,74	99,74	99,74	99,74	99,74	99,74	99,74	99,74	99,74	91,56	92,07	92,07	82,35
KF410630 *Trichuris* sp. *P. hamadryas*	100	100	99,49	99,49	99,49	100	100	100	100	100	91,82	92,33	92,33	82,61
KF410629 *Trichuris* sp. *P. hamadryas*	99,74	99,74	99,74	99,74	99,74	99,74	99,74	99,74	99,74	99,74	92,07	92,07	92,07	82,86
