# Supplementary material for: New Insights on Tools for Detecting β-Tubulin Polymorphisms in Trichuris trichiura Using rhAmpTM SNP Genotyping
Source: Animals (Basel). 2024 May 23;14(11):1545. doi: 10.3390/ani14111545 (PMC11171370; doi:10.3390/ani14111545)
Supplement: Supplementary file 1 [file animals-14-01545-s001.zip › Supplementary Table S2b.pdf]

Table S2b. Continuation of Table S2a. Intra-specific and inter-specific similarity observed in β-tubulin partial gene sequences in *Trichuris* species isolated from different hosts. Values are given in percentages (%).

	OQ446481 *Trichuris* sp. from *H. cristata* TPEF15	KF410628 *T. trichiura* from *H. sapiens*	KF410627 *T. trichiura* from *H. sapiens*	KF410626 *T. trichiura* from *H. sapiens*	KF410625 *T. trichiura* from *H. sapiens*	KF410624 *T. trichiura* from *H. sapiens*	KF410623 *T. trichiura* from *H. sapiens*	MW403705 *Trichuris* sp. from *M. fuscata*	MW403706 *Trichuris* sp. from *M. fuscata*	KF410634 *Trichuris* sp. *P. hamadryas*	KF410633 *Trichuris* sp. *P. hamadryas*	KF410632 *Trichuris* sp. *P. hamadryas*	KF410631 *Trichuris* sp. *P. hamadryas*	KF410630 *Trichuris* sp. *P. hamadryas*
KF410628 *T. trichiura* from *H. sapiens*	82,61													
KF410627 *T. trichiura* from *H. sapiens*	82,35	99,74												
KF410626 *T. trichiura* from *H. sapiens*	82,10	99,49	99,74											
KF410625 *T. trichiura* from *H. sapiens*	82,35	99,49	99,74	99,49										
KF410624 *T. trichiura*	82,86	98,98	99,23	98,98	99,49									

from *H. sapiens*														
KF410623 *T. trichiura* from *H. sapiens*	82,61	99,23	99,49	99,23	99,74	99,74								
MW403705 *Trichuris* sp. from *M. fuscata*	82,10	98,72	98,98	98,72	99,23	99,23	99,49							
MW403706 *Trichuris* sp. from *M. fuscata*	81,84	98,47	98,72	98,47	98,98	98,98	99,23	99,23						
KF410634 *Trichuris* sp. *P. hamadryas*	82,61	99,23	99,49	99,23	99,74	99,74	100	99,49	99,23					
KF410633 *Trichuris* sp. *P. hamadryas*	82,86	98,98	99,23	98,98	99,49	99,49	99,74	99,23	98,98	99,74				
KF410632 *Trichuri*	82,61	99,23	99,49	99,23	99,74	99,74	100	99,49	99,23	100	99,74			

s sp. *P. hamadryas*														
KF410631 *Trichuris* sp. *P. hamadryas*	82,35	98,98	99,23	98,98	99,49	99,49	99,74	99,23	98,98	99,74	99,49	99,74		
KF410630 *Trichuris* sp. *P. hamadryas*	82,61	98,72	98,98	98,72	99,23	99,23	99,49	98,98	98,72	99,49	99,23	99,49	99,74	
KF410629 *Trichuris* sp. *P. hamadryas*	82,86	98,98	99,23	98,98	99,49	99,49	99,74	99,23	98,98	99,74	99,49	99,74	99,49	99,74
